# Leaves from the Diary of a Patient Confined in Hanwell Asylum

**Published:** 1856-10-01

**Authors:** 


### Art. IV.—LEAVES FROM THE DIARY OF A PATIENT, CONFINED IN HANWELL ASYLUM.

[The following diary has been placed in our hands by the writer, who was confined in the Middlesex County Lunatic Asylum for six months. During a portion of that period he kept a daily record of the treatment to which he was subjected. The writer speaks well of the Asylum and of those officially connected with it, although he feels that he was unjustly sent and detained there. As giving some idea of the internal economy of this national establishment, the following extracts may prove of interest to our readers.]

*Monday, 12th January,* 185–.—Brought in a cab here (Hanwell Lunatic Asylum); stripped of my clothes, and enforced to take a warm bath. Examination of my person, to see, I presume, what marks, if any, I had about me, so that my body might be sworn to, in case I drowned, or hung myself—no great improbability of either event taking place, considering what I had latterly been subjected to.

After my bath, I was allowed a rather wide run over the building—a wonderful one, truly. Having arrived here about half-past twelve o'clock a.m., and the dinner hour in this vast establishment being one p.m., I had not been an inmate long before dinner was discussed. It comprised soup, meat, and vegetables, with about one-third of a pint of—not over-strong, but, what to me is more desirable—*genuine* table ale. After dinner I was taken before Dr. B., the resident physician, who listened, apparently with considerable interest, to a somewhat lengthy epitome of my checkered history during the last ten years. I was then conveyed to my future "ward," that is, the particular room or apartment where lunatics of my presumed class were confined, or rather where we took our meals together, and familiarised. And here I must say that I was much surprised to find men who, to me, for the first few hours of our associating together, appeared in every respect to be in full possession of all their rational faculties. My thoughts thereupon were akin to these—If these men be really mad, why, then I do not wonder at others deeming me so, and while that supposition exists, here, doubtless, I am likely to remain. This was not a pleasing idea by any means, but "*nil desperandum!*" At five minutes before five p.m., the bell tolled for chapel. It was perfectly optional on a patient's part whether he chose to attend the service or no. Indeed, I may say here that the system of "non-restraint," under which this vast asylum is conducted, is most consistently carried out. I attended the evening service, which commenced with singing a short hymn; then the minister read two or three prayers from the Church of England Common Prayer Book, and then was read a chapter in the New Testament; the service occupying about twenty minutes. On my return to my "ward," *tea* was served: each patient was supplied with about two-thirds of a pint, very little milk, less sugar, no bread or butter; in fact, this is not looked on as a meal at all, but is altogether supplementary to the asylum dietary, and considered a luxury and an indulgence. At seven o'clock, however, "supper" was served; this was a tolerably substantial meal. It consisted of nearly half-a-pint of the before-mentioned table ale, a thick slice of very excellent bread—yesterday's—and about an ounce and a half of tolerably good cheese. This being disposed of, I was conducted to bed. Bed!- imagine, seven o'clock p.m.! Considering, however, that I should gain nothing to my advantage by any expression of dissent, I philosophically bottled my indignation, and unhesitatingly suffered myself to be conducted to my "dormitory." But I cannot help here frankly confessing that my pent-up feelings approximated their explosive point when I found that four other—to use a mild term, say eccentric, gentlemen—were to sleep in the same apartment with me. The "attendants" (there are no *keepers* here, *they* having all disappeared about the same period when the *whip, irons, strait waistcoats,* and other comforts of the like nature, thank God, ceased to be!)—the attendants kindly informing me that the reason we were to sleep in company, arose from the fact that one or two of my "friends" had a foolish *penchant* for hammering their heads against the walls, and that it was found that "company" restrained that exceedingly low and silly behaviour. This communication, I need scarcely say, was not likely to render me more inclined to sleep than I already was, although the attendant, doubtless, imagined the contrary. I was ordered to tie up my clothes in a bundle, and place them outside my door, along with the respective suits of my sleeping companions, and we were then locked in. I do not think I have before said, that after I had taken my bath, when I first entered here, my own clothes were taken from me, and an old suit of "grey" given me in their stead; this being one of the "rules" of this establishment. But to proceed. The attendant, after locking us in, bade us good night through an eyelet hole in the door, and we were left for sleep, but this balmy comforter was a long, long time before he visited me; and I laid on my little bed—we each had a separate one—tossing restlessly about, and not altogether free from imagining that probably some one of my eccentric "friends" might feel inclined to lay—in the penny-a-liner's language—"violent hands" on me. I also sighed, and could not help thinking of the train of singular circumstances which had —O, how unjustly! consigned me here as a madman; and then I thought of my home, from which I had been pitilessly torn, of my dear wife, to whom I have been so few years—*not three* —married, and of my child! Sleep, at length, happily, overtook me, and without dreaming (I have never been a dreamer), after a refreshing night's rest I was awoke the next morning at six o'clock. This is the hour when the attendants of each "ward" are ordered to unlock the doors of their respective patients. Having dressed and washed, I amused myself with a book till the morning chapel bell rang, about "eight;" and with perhaps a hundred, or a hundred and fifty others, I attended the service.

*Friday, 16th January.*—Being by this time looked upon by the authorities here as possessing at least *some* of my faculties, I was inducted to an assistantship in "the store" here, where my duties comprised the weighing out of tea, sugar, tobacco, snuff, &c. This was to me a desirable occupation, for I have always liked to be employed; and thankful I was for so soon experiencing one of the rules of the excellent system under which this Asylum is conducted; that is, the employing as much as possible its unfortunate inmates, in doing something useful to the institution itself; and thus, not only amusing some of my "friends" and myself, but saving a trifle to the county. The superintendent of "the store," Mr. C., treats me with really considerable kindness and attention, and what is more congenial to my feelings still, consoles me by intimating that in all probability my detention here will be but exceedingly limited. He requests me also to take whatever tobacco, snuff, or other little luxury I may fancy, and I had an excellent lunch this morning —to be continued, of course, on good behaviour—of superior bread-and-cheese, washed down with that very scarce beverage in the "metropolis"—*unadulterated* porter! In the afternoon of to-day, I was visited by my dear wife, and allowed to walk with her, unaccompanied by attendants, for two or three hours, on the very pretty greensward which charmingly ornaments the somewhat extensive front of this Asylum.

*Saturday, 17th January.*—A most beautiful and inviting morning: such a one when we envy the freedom of the happy birds, and should we unhappily be prisoners, long to burst our prison-bars and revel in the luxury of liberty. About eleven o'clock, having previously obtained leave of absence from my master *pro tem.*, Mr. C., I was allowed to take rather a wide promenade round the extensive grounds which surround this noble building, and which grounds are cultivated by efficient "hands,"

together with the not incontemptible assistance of such of the patients who are sufficiently capable, and are so desirous of helping in rearing the vegetables requisite for the uses of this vast establishment. I was with a "company" or "gang" of about twenty, and one attendant, as a sort of overlooker, who, besides looking after the safety of his pupils, gave them instructions what work to go about, whether "hoeing," "weeding," or whatever the particular labour required. Quickly noticing that we were now in a field only separated from the public road by a hedge, which I felt a very little exertion on my part would carry me over, I artfully and gradually drew myself from my fellow-prisoners, and pretending to be examining the different vegetables that surrounded me, managed, after a few minutes, to escape the eyesight of the attendant, who had no suspicion of my intention, and quickly leaping the not very high or difficult hedge, found myself in the London road. Free! A cart coming along the road from the direction of the last-named place, I soon persuaded the lad driver to let me accompany him away from Hanwell, asking him at the same time if he thought me mad! He unreservedly answered, No! and effected my escape from the vicinity of the Asylum by concealing me under some horse-cloths or things of the kind at the bottom of the cart, and we soon jogged along, thank God, happily enough. I did not attempt to show myself, as I had the Asylum dress on, and was, of course, afraid of being recognised by some one, and sent back to my old quarters. After a ride of, I presume, some three or four miles, the lad acquainted me with the fact of his nearing his destination. Leaping, cheerfully, therefore, from the cart, and much thanking him—in *words*, "for *silver and gold had I none*"—for his kindness in conveying me away safely so far, I bade him adieu, and at once proceeded to complete my escape; for I was still within what I thought much too easy a distance from Hanwell to consider myself free from the danger of being re-taken. Walking and running on, therefore, I at length came to a canal, along whose towing-path I at once made up my mind to proceed, imagining that by a circuitous direction it made its way into London. And this, I have subsequently ascertained from the attendants here, is the fact, it being a part of the well-known "Regent's Canal," which runs through this part of the country on its way to the metropolis.

At length, thinking myself somewhat safe, I less cautiously began to look around me, when all at once, O horror! my eyes were completely fascinated—I don't think I could pick out a word more expressive—at the abrupt appearance of one of the attendants of the Asylum, in his blue frock-coat and brass buttons, running in a parallel direction, and within a stone's throw of me.

Noways intimidated, however, and thinking I could perhaps outrun him, I accordingly "picked myself up," as sporting men say, and ran with all my speed. Ahead of me, unluckily, and standing on the very towing-path on which I was running, were six or seven ill-looking, skulking fellows, and to whom, immediately he saw them, my pursuer vehemently shouted to stop me. There were only now, then, two alternatives,—either to deliver myself quietly into the hands of the attendant, or to "*take the water.*" Had the chace have happened on a warm day in summer, I believe I should have chosen the latter impulse (for I can swim), but as it was a bitter day in January, I surrendered at discretion. Within an hour subsequently I was once again lodged in safety in my "ward."

*Sunday, 18th January.*—About ten o'clock this morning, by direction of Dr. B., I was "transplanted," if I may be permitted to use such a word under present circumstances, from "Ward No. Eleven" to "Ward No. Six"—a "*Refractory*" one. This, it seems, is a punishment for my daring attempt at escape. I am glad to find, however, that although the patients are much more boisterous, and, possibly, more dangerous, than my first fellow-prisoners, the "attendants"—and a great deal rests with them—are more quiet and gentlemanly than those in my previous "ward;" and I am made by them as comfortable as their duties, and the circumstances under which I am detained here, admit of.

*Thursday, 29th January.*—To-day I have been examined by the visiting justices, magistrates who periodically visit and form a "board" here, to see and discharge, if convalescent, the patients who are sufficiently well to appear before them. The chairman of the board to day, Mr. W., addressed me in an exceedingly kind manner; and further said, that he hoped and believed I should very soon appear before them again to ask for my discharge. The clouds are now, I think, beginning to break, and I am almost tempted to believe that the day of my freedom from detention in this asylum, as a madman, is not far distant. O may my presentiment be realized!

*Saturday, 31st January.*—This morning I was supplied with a new suit of really very decent looking grey clothing. On putting them on, I found there was plenty of room left me to grow stouter in; and which, if I prove no exception to the general rule of all those who become inmates here, in the course of a very few weeks I shall. Such seems to be really the case, according to all accounts here, a very short space of time being sufficient to considerably increase the bulk of the inmates of this asylum. In the afternoon of to day I was removed from "Ward No. Six" to "Ward No. Four," where I now write this. This

"removal," it seems, was to accommodate a new patient, who took possession of my old quarters immediately I vacated them for these.

*Sunday, 1st February.*—I like my new "ward" as much better than "Ward No. Six," as the latter was preferred by me to "Ward No. Eleven," the one in which I was first located. I pen these lines in my *bed-room.* I have *one here to myself;* and which to me is infinitely more agreeable than "the associative principle" I have heretofore been used to. I am happy in finding also that the "attendants" in this ward are very kind and considerate, and really treat me with some little deference, as though they considered me somewhat above the common lunatic they are accustomed to. I cannot help repeating how thankful I am to that great Giver of all in thus letting me taste happiness, even in a lunatic asylum. My dear wife visited me again to-day, by the kind permission of Dr. B.,—Tuesdays and Fridays being the regulation days,—and brought with her our darling Harry. I am sure the poor child did not know me at first, so much do these pauper clothes and short cut hair alter my appearance. I have a slight revenge for the latter, however, in allowing my beard to grow, Our dear boy soon found I was "father;" and we were not long before we were gambolling on the floor, as was my wont before our separation. I accuse myself, since my wife has gone home, of having been harsh and unfeeling to her to-day. I hope God will forgive me for this unbridled tongue, and which, unhappily, I too frequently make others feel the poignancy of. The company of my wife and little one gave me unalloyed pleasure; and I romped with my dear boy with as much enjoyment, ay, as if I were a child myself again!

*Wednesday, 4th February.*—I do not find my new "ward" *quite* so comfortable as I did a day or two ago; nor are my spirits, I am sorry to say, so exuberant as they have hitherto been since my detention. The awful blasphemy continually being expressed in this "ward," by one or another of its unhappy demented inmates, is to me truly horrifying; and I hope I shall shortly be removed to some other "ward," where my fellow-patients are milder in their language and behaviour. I have mentioned my wish to the good doctor, who has promised to do all he can for me.

*Saturday, 7th February.*—I have now been in this "ward," No. Four, a week. I shall be very glad to get a change, which I am told I may now expect in a day or two. In this "ward" we certainly have some dreadful characters. Among them is an old and toothless man, nearly eighty years of age, who has formerly been, I understand, a keeper of madmen himself! He often mumbles about putting those he is quarrelling with into *double irons*, thereby evidencing, plainly enough, his former occupation. From what I can see of him—and perhaps I have no right to form any such opinion, as they nearly all tell me here that I, myself, am but a madman; and God knows I may be; I have had troubles enough at least to render me so—I should opine that he has been a very vile and unfeeling fellow, if no worse; and I am inclined to look upon his detention here as God's justice this side of the grave. He is so violent at times, that it takes three or more of the attendants to overpower him, although he is at so advanced an age. After which, as a punishment, they confine him in the water-closet till he becomes a little less noisy. Each night in this ward, from the hour we are locked up in our respective bed-rooms—seven o'clock—till half-past nine or ten, the place is one continual and horrid din — *a hell upon earth*—holloing, hooting, shouting, kicking, swearing, singing, whistling, shrieking, and hammering at cell doors; and although each poor demented wretch is separately and securely confined under the best and safest materials, I confess, even in spite of the alleged security of their fastenings, I am sometimes fearful lest one of them should burst his door open and come and tear me to pieces: their united noises, blending unharmoniously together, producing a chorus of so extraordinary a character, that my pen falls very far short of expressing. I must not omit to mention here another member of this eccentric "choir;" an individual who rejoices in the name of Kitty Fisher, who, when the noise is at its highest pitch, and I am nervously expecting every moment to hear six or seven of the doors burst open, with their respective reports like so many pieces of ordnance, in chimes Mrs. Kitty Fisher with an extremely clever imitation of that far from soporific sound, the loud and continued braying of a healthy young jackass!

*Thursday*, 12*th February*.—Three days ago I was moved from "Ward No. Four"—the one in which I last wrote anything in this Diary—to the one I now occupy, "Ward No. Nine." This ward contains upwards of fifty patients, and is named a *convalescent* "ward." In fact, there are more men here than are pleasant to me; and I find it at times quite a difficulty to enter anything in my diary, there being now in my presence fifteen or sixteen lunatics, some of whom are so curious, or so impudent, as to be slyly peeping over my shoulder at this very moment, and trying—but I flatter myself unsuccessfully—to decipher my somewhat illegible penmanship. I do not, however, evince any resentment at their rude behaviour, knowing the terrible affliction under which they suffer. I may perhaps enter the fact here as rather remarkable, that although I have been an inmate of this asylum but a few weeks, I have grown fat, and am told by my friends, who visit me occasionally, and I think so myself, that I have a considerably healthier and better appearance than that which I possessed when I first came here. So much for country air and "pauper dietary." All my acquaintance well know I have never been distinguished for anything like obesity.

*Friday, 20th February.*—My dear wife visited me again to-day. I do not remember ever seeing her look more charmingly. She was very pale, it is true; but that only rendered her in my eyes the more interesting; and I dare say, had she appeared ruddier, I should have charged her with not thinking much of me. I can now better appreciate that sweet sentiment expressed by Scott in "Marmion"—

> "When pain or anguish wring the brow,
> A ministering angel thou!"

Certainly my dear wife's affection seems much strengthened since my incarceration here. And, thank God, she ascertained from Dr. B—— this afternoon his opinion that if I only went on as well as I do at present, he thought he might assure her that he should give his sanction to my discharge in a few days. I thank God most heartily, but humbly, who has thus answered my daily and nightly prayer.

*Tuesday, 24th February.*—To-day I have been visited by my brother and sister, and her good and kind husband, Mr. C——. They brought me several very acceptable little presents, which much tend to alleviate the somewhat monotonous existence necessarily attendant on my detention here. And I cannot help repeating how thankful I am to the Great and Good Giver of All, who thus is ever mindful of me, and beneficently provides me, even in a madhouse, with not only every necessary, but even many luxuries. Oh, that I may be permitted, ere I die, to do some good work, however inadequately, in part payment for this supreme beneficence!

*Saturday, 28th February.*—I am again brought into a "Refractory Ward." This was done by direction of Dr. B——. I had just finished a letter to my dear wife, and was about putting a postage stamp on the envelope, when one of my fellow-patients—certainly a man who knew entirely what he was about, and, in my humble opinion, possessed more intellect than very many "rational" persons outside of this asylum—grossly insulted me. I shall not sully this page, nor pollute my lips, by repeating his very offensive language. Words naturally arose between us, when the Doctor happening to approach—and who, I am sure, could not have heard the *whole* of the altercation between us, or he would have acted otherwise—ordered me at once into this "Refractory Ward," and left the aggressor in my late ward, who laughed at poor me as I was conducted here. O, my God! give me patience to bear with these wretches, who thus inconsiderately trample on a poor man's feelings!

*Sunday, 29th February.*—To-day, by Dr. B.'s order, I have returned to my late ward, the "Convalescent," "No. Nine," and from which I was so abruptly, and I think I may say inconsiderately, removed on Friday morning. I believe I may conclude that I have been brought back here, after being in the "Refractory Ward" but for so short a time, by an act which, to many men's minds, I must frankly confess, would really assume the appearance of none other than a madman's. It was this. During the whole of yesterday I was extremely indignant that I should be again confined in a "Refractory Ward" so soon after the doctor had intimated my convalescence and early departure from this horrid place. This, coupled with a very loving letter from my dear wife, wherein she expresses her joy at our soon meeting, and living together again as formerly, made me determined, if possible, to anticipate our felicity by breaking out of my prison-house, and flying at once to her. Having arranged my plan, therefore, immediately after tea yesterday I commenced carrying the same into execution. I first of all broke off from a somewhat large iron soup-ladle its nice long handle, about two feet and a half in length, with a stout hook at its end, most convenient, as I soon found, for picking locks, and certain other, I suppose I must say, nefarious purposes. This was certainly a capital instrument, and I at once essayed to ascertain if with it I could pick the lock of the door of the cell wherein I that night was to sleep. This unfortunately I found was impracticable, the lock being much too good a one to be tampered with in that manner. I thereupon altered my plan, and commenced weakening the socket into which the bolt was thrust by the action of the key, the lock being fastened from the outside. A half hour's picking at this left it in such a condition that I felt little doubt but that a moderate force from the inside, and such as I could command, would release the door from its fastening. Having accomplished this, and it being then near our supper time, that meal was shortly disposed of, and at seven o'clock as usual, with the other inmates of the ward, I was, as the "attendant" doubtless believed, securely locked up for the night. After waiting with all the patience that I could summon for the occasion, at length the "coast was clear." The patients were all fastened within their respective "dormitories," and the attendants, being now *off* duty, were smoking their pipes and drinking beer, fortunately, in a part of the asylum at too great a distance to hear anything of my somewhat alarming operations. The bedsteads in these "Refractory" wards have what is here called

"a stretcher." This is a wooden frame, about six or seven feet in length, and perhaps from three to three feet and a-half in width, and in depth, some six or more inches; the whole very strongly secured together, and, still further, a very stout iron bar or pin, running across the middle, augmenting its strength, and likewise available as a handle, in moving it from place to place. Across this frame a stout canvas is tightly drawn—drum fashion—on which the mattress is laid, and a very good sleeping apparatus it makes—of course, with a complement of the other usual requisites, such as blankets, sheets, &c. Well, using this stretcher as a sort of battering-ram, after a few smart blows at my cell door with it, I very soon had the pleasure of seeing it fly open. I then began tearing up my counterpane in strips, tying them together as I did so, contemplating breaking a window of the gallery in which I then was—the third story—and with the assistance of my *impromptu* rope, descending to the yard of the asylum. This, I soon found, however, was a work of too much time, and again altered my plan as follows:—I first sought the poker, in order, if possible, to break some thick iron railings which prevented my descent by a staircase into the floor below. Around the fire-places in these "refractory" wards is fixed a stout iron fence, or net-work, to prevent accidents from the patients burning themselves, and also to keep from their use the "fire-irons," which, being very large and heavy, would make truly formidable weapons of offence in the hands of any who laid hold of them for such purposes. This net-work of iron was secured by a considerably strong padlock, and which I only succeeded in forcing open after a great amount of exertion, and at length, luckily, secured the poker, and a splendid fellow he was: about four feet and a-half long, and very nearly, if not quite, an inch in thickness. I then attempted to break the iron railings which I before mentioned as preventing my descent into the gallery on the next floor, but soon found, to my chagrin, they were much too strong for me. After a few unsuccessful blows, therefore, I relinquished the attempt. It was now nearly ten o'clock, and I was walking up and down the gallery ruminating on the next best course to pursue, when, all at once, I heard one of the "attendants" open a door at some short distance from me. To rush into my cell was but the work of a moment or two; unfortunately, I did not, in my hurry, close the door after me sufficiently close to escape his keen observation. He tried it, and immediately saw how matters stood. My "game" was now, I thought, "up;" I had the poker in my hand, and might have struck him, had I been so minded; but instead of doing so, thank God, I let him take the awkward plaything quietly out of my hand, and he then left me, doubtless to publish the fact to his "mates" that I was "at large." I now made up my mind to parley with Dr. B. before I gave myself up, and expressed my desire to see him to the attendant, as he left me to fetch, as I thought, further assistance. Immediately I was by myself, I grasped the shovel—a weapon equally as formidable as the poker I had let the attendant take from me.

I had but just time to chuckle over my prize, when in entered the doctor himself, with about eight or nine of the stoutest "attendants." Shouldering my shovel in as alarming a manner as I could, and feeling naturally excited, I swore that I would knock down the first one who attempted to lay hands on me. The men, who stood at a respectable distance from me, knowing my determination and spirit, were really alarmed, and therefore, along with the doctor, began to parley. In the meantime other men had been sent round the Asylum so as to encircle me and come upon me from behind. The latter soon making their appearance, and seeing myself entirely in their power, and certainly having no desire to injure any of the attendants, who I of course knew were only doing *their* duty, I made no further resistance, and they soon closed upon me, and using me not unkindly, soon stripped me entirely naked, to see, I suppose, that I had no weapon about me, and giving me back my flannel waistcoat and shirt, thrust me into a "padded room." A bed was hastily made therein for me, the door was secured, and I was once more safely under lock and key.

---

